# Ichthyosis Congenita, Harlequin Type: A Fatal Case Report

**DOI:** 10.7759/cureus.3524

**Published:** 2018-10-30

**Authors:** Amber Tahir, Syed Maaz Tariq, Syed Ali Haider, Mohammad Hasan

**Affiliations:** 1 Internal Medicine, Dow University of Health Sciences, Karachi, PAK; 2 Internal Medicine, Jinnah Sindh Medical University, Karachi, PAK

**Keywords:** harlequin icthyosis, ichthyosis congenita, congenital anomaly, case report, pakistan

## Abstract

Harlequin baby is rare and it is the most severe kind of congenital ichthyosis. It manifests as severely keratinized skin with an autosomal recessive inheritance. Incidence of this disease is 1 in 300,000 live births. We report a new case of harlequin ichthyosis (HI) from Pakistan to contribute to the collective knowledge of this condition. HI is associated with *ABCA12* gene mutation; hence, genetic screening and counseling to susceptible parents must be considered.

## Introduction

Icthyosis congenita or harlequin icthyosis is a rare, dreadful condition with an incidence of 1 in 300,000 live births [1]. The fetus born with this condition is characterized by severe erythrodermic ichthyosis and desquamation of the epidermis that gives the baby a characteristic appearance at birth [2-3]. It is inherited by autosomal recessive mode of inheritance giving rise to the notion that consanguinity between parents has a role to play in its etiology [4].

## Case presentation

A 34-year-old female, Gravida 4 Parity 3+0, was seen in the emergency room of a tertiary care hospital, in Karachi, with labor pains. She had all previous uneventful vaginal deliveries with healthy and alive babies. The baby was the fourth outcome of a consanguineous marriage, the couple being maternal first cousins. The parents did not report any history of repeated miscarriages/stillbirths, genetic anomalies in the elder children, or any inherited skin disorder. It was an unbooked case. She had no antenatal record available. 

Abdominal examination revealed the height of fundus roughly equivalent to almost 36 weeks of gestation and the fetal head engaged. The labor progressed spontaneously. A female baby of weight 1.8 kg was delivered with Apgar score 7 and 8 at 1 and 5 minutes, respectively.

**Figure 1:**
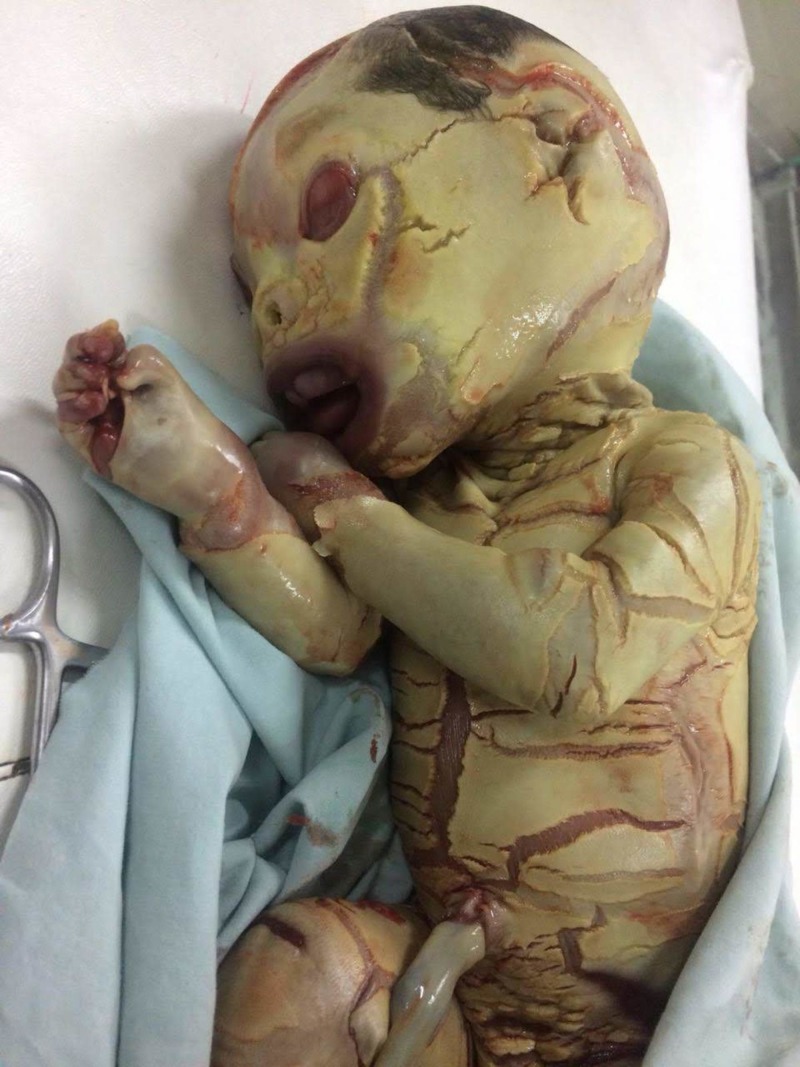
Signs of harlequin ichthyosis at the time of birth.

At the time of birth, the body of the neonate was covered with armour-plate-like, thick and widespread yellowish scales; between these keratin layers of scales were erythematous fissures splitting the scales and extending deep into the dermis. The scales and fissures covered the body in a diamond-like pattern and were marked in the flexures. All limbs and digits were rigidly semi-flexed. Other evident features included ectropion, eclabium with a fixed and wide-open mouth, flat nasal bridge, and rudimentary external ears. The eyebrows and eyelashes were absent; however, scalp hair was evident. All natural orifices were patent (Figure 1).

The baby was evaluated by the pediatrician and the clinical diagnosis of harlequin ichthyosis was made. The family was counseled and the baby was shifted to the neonatal intensive care unit (NICU) where she was kept in a humidified incubator. Although the baby had adequate sucking reflex, in order to prevent any incidence of aspiration, she was started with parenteral nutrition, intravenous antibiotics and continuous positive airway pressure (CPAP) was delivered via Bubble-CPAP. Skin was cleaned with normal saline. Liquid paraffin and emollients were gently applied to the whole cutaneous surface every two hours. Topical antibiotics were also added to the skincare. Antibacterial eyedrops and sterilized cotton eye pads were applied. The baby developed respiratory distress within a few hours of birth, despite the supportive management. The baby died on the second day of life due to respiratory distress. The parents were psychologically supported, the disease spectrum was explained to them, and genetic counseling was recommended for future pregnancies.

## Discussion

Harlequin ichthyosis, also known as ichthyosis congenital or keratosis diffusa foetalis, is an extremely rare genetic disorder. It is characterized by severe erythrodermic ichthyosis and desquamation of the epidermis that gives the newborn a characteristic and alarming appearance. Other findings reported with this presentation are severe ectropion, eclabium, alopecia, digital contractures which may require a surgical consult and if not dealt with timely, may result in gangrene necessitating amputation, growth retardation, immature nostrils, and external auditory meatus [2-3]. It is inherited by autosomal recessive mode of inheritance which also suggests the role of consanguinity among parents as a contributing factor. The affected individuals are homozygous for non-sense mutation in *ABCA 12* gene (adenosine triphosphate-binding cassette transporter, subfamily A, member 12) on chromosome 2q33-q35 resulting in premature termination of protein translation [4]. The *ABCA 12* gene plays a crucial role in transporting lipids to various body cells. These lipids are essential for physiological development of the epidermis [5]. Almost half of the children born with this condition do not survive due to dehydration, sepsis, respiratory failure, hypothermia, hypoglycemia, and renal failure [6].

Perinatal diagnosis is important. Examination of amniotic fluid cells and an ultrasound, mainly of the fetal mouth particularly at the 17th week of pregnancy have shown to yield decisive results [7]. Additionally, a detailed account of family history, consanguinity between parents, information about previous pregnancies, and whether or not other children had any inherent dermatological condition, are of importance. Postnatal diagnosis includes a skin biopsy which would presumably demonstrate structural abnormalities of lamellar granules and epidermal keratin expression. Usually the gross appearance of fetus is sufficient for diagnosis.

The nature of complications and its associated co-morbidities warrants a multi-disciplinary approach. Initial management includes a humidified incubator for encountering transcutaneous water loss, physiotherapy, analgesia for painful deep fissures, proper infection control, and maintenance of nutritional status. A comprehensive case series comprising 45 cases suggested oral retinoids, preferably acitretin, which works by shedding the hyperkeratotic encasement causing a phenotypic switch aids for increasing the survival. The choice of acitretin is supported by a favorable side effect profile and shorter half- life [8]. For the first month of life, until the deep fissures heal, sepsis remains a threat and is unfortunately the leading cause of death in these patients [2]. Despite the risk of sepsis, quite interestingly there are no data available which supports antibiotic therapy in these patients and in a case reported by Gunes et al., the patient died on day 21 after being administered antibiotic treatment [9].

## Conclusions

Harlequin icthyosis is a rare genetic condition of the skin. Genetic counseling, genetic screening, and prenatal diagnosis must be advised to susceptible parents. Supportive therapy remains the mainstay of management; however, these neonates do not have a very good prognosis.
